# Multiple Brain Abscesses in an Immunocompetent Patient With Factor V Leiden Mutation

**DOI:** 10.1177/2324709616683724

**Published:** 2016-12-01

**Authors:** Saeed Zubair Zafar, Najwa Pervin, Sukesh Manthri, Mukul Bhattarai

**Affiliations:** 1 Southern Illinois University, Springfield, IL, USA

**Keywords:** cerebral abscesses, immunocompetent, factor V Leiden

## Abstract

Multiple brain abscesses in an immunocompetent patient is a challenging clinical problem in the medical world despite advances in imaging techniques, laboratory diagnostics, surgical interventions, and antimicrobial treatment. It is a clinical entity that typically tends to occur in the presence of known predisposing factors. Clinicians seek to determine the potential risk factors responsible for the development of brain abscess because it is very crucial for management of this life-threatening condition. At times, like in our case, there are clinical situations where it is difficult to reveal any traditional risk factors. We report a case of multiple brain abscesses in a 51-year-old female with a past medical history significant only for factor V Leiden mutation, and deep vein thrombosis on chronic anticoagulation. She underwent thorough evaluation but no predisposing factors were found. Based on our extensive literature review, this is the index case of multiple brain abscesses in a patient with history of factor V Leiden mutation and the absence of any conventional risk factors. We also postulate a possible mechanism of infection in such patients.

## Introduction

Despite advances in imaging techniques, laboratory diagnostics, surgical interventions, and antimicrobial treatment, brain abscess remains a challenging clinical problem with substantial case fatality rates.^1^ This infection can result from either contiguous spread or hematogenous dissemination of pathogens into the central nervous system. The pathologic mechanism of development of brain abscesses depends on predisposing conditions. The commonly identified predisposing risk factors for such infections are underlying disease (eg, infection with the human immunodeficiency virus [HIV]), a history of treatment with immunosuppressive drugs, disruption of the natural protective barriers surrounding the brain (eg, due to an operative procedure, trauma, mastoiditis, sinusitis, or dental infection), or a systemic source of infection (eg, endocarditis or bacteremia). In addition, congenital heart disease, meningitis, and certain procedures such as esophageal dilatation are also associated with multiple abscesses in brain parenchyma. The multiple brain abscesses in patients in the absence of identifiable risk factor are very rare and have been reported sporadically.

## Case Description

A 51-year-old Caucasian female with a past medical history significant for factor V Leiden mutation, deep vein thrombosis on chronic anticoagulation, and chronic stable anemia secondary to menorrhagia due to uterine fibroid presented to the hospital with altered mental status, fever, and headache of 1 day duration.

**Figure 1.**
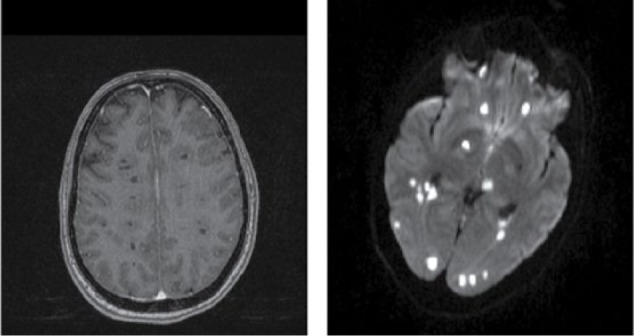
MRI images from day of admission. Post contrast T1 weighted image (left); diffusion weighted image (right).

At admission, she was febrile with a temperature of 103°F, tachycardic, and confused. Her physical exam was remarkable for neck rigidity, decreased level of alertness, and disorientation to place and time. She emergently underwent lumbar puncture and was started on empiric therapy with vancomycin, ceftriaxone, and ampicillin. Magnetic resonance imaging (MRI) brain was done that showed innumerable focal parenchymal lesions involving cerebral hemispheres, deep gray matter, midbrain and both cerebellar hemispheres (Figure 1). The majority of the lesions measured between 5 and 10 mm in diameter with the cerebral and cerebellar hemisphere lesions distributed predominantly at the gray-white junction with additional periventricular and basal ganglia lesions bilaterally.

Cerebrospinal fluid (CSF) analysis showed >20 000 white blood cells with 95% neutrophils, glucose 1 mg/dL, and CSF protein 339 mg/dL. The blood cultures grew *Pepto-streptococcus* and *Streptococcus intermedius*. Based on her blood cultures, her antibiotics were optimized to vancomycin, meropenem, and an antifungal voriconazole was added. A stereotactic brain biopsy was performed after a week of antibiotic treatment that showed brain abscess; however, no organism was isolated from the specimen. Transesophageal echocardiogram with bubble study showed no evidence of vegetations, valvular lesions, or patent foramen ovale. Her history was reviewed extensively to determine the source of infection. The patient was not a drug abuser, and her urine toxicology was negative. The patient denied having any recent dental infections, dental work, sinus infection, maxillofacial surgery, or head trauma prior to development of symptoms. HIV screening and CSF analysis for toxoplasmosis were negative. Central nervous system (CNS) lymphoma was ruled out based on the acuity of symptom development and lack of B symptoms.

**Figure 2.**
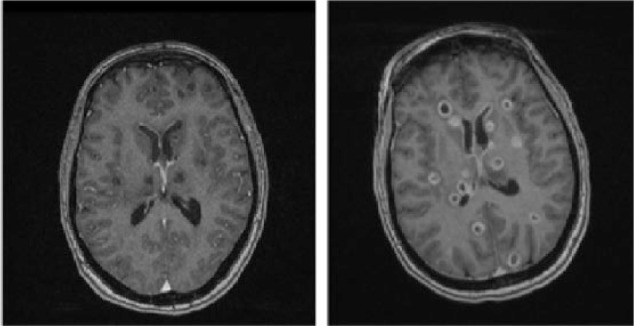
Contrast MRI on left from day of admission shows little if any peripheral enhancement. Image on right, obtained 9 days later, shows interval organization of parenchymal abscess with rim enhancement.

Repeat MRI 9 days later (Figure 2) showed enhancement of the ring enhancing lesions without any new lesions. She gradually improved and was later transferred to skilled nursing facility for rehabilitation where intravenous antibiotics were continued for 6 more weeks. On follow-up at clinic, repeat CT scan showed significant reduction in size of lesions, from 5to 10 mm to less than 3 mm. Her cognitive, sensory, and motor functions returned back to baseline.

## Discussion

Brain abscess is defined as a focal infection within the brain parenchyma, which starts as a localized area of cerebritis, which is subsequently converted into a collection of pus within a well-vascularized capsule.^2^ Brain abscess can be caused by bacteria, mycobacteria, fungi, or parasites (protozoa and helminths), and the reported incidence ranges from 0.4 to 0.9 cases per 100 000 population. Rates are increased in immunosuppressed patients.^3,4^ Most frequently isolated microorganisms are *Viridians streptococci, Staphylococcus aureas*, gram-negative bacilli, and anaerobes.^5^ In many of these cases, *Streptococcus intermedius* and *Pepto-streptococcus* were isolated from CNS lesions. These streptococci are common inhabitants of the mouth, nasopharynx, gastrointestinal tract, and vagina, with an isolation rate of 15% to 30%.^6^

In a general population, bacterial brain abscesses can develop from 3 sources: first, because of the spread of infection from a pericranial contiguous focus (such as the sinuses, middle ear, or dental infection) in 25% to 50% of cases; second, from a distant focus of infection (such as a lung abscess or empyema, endocarditis, skin, or the intraabdominal cavity), resulting in hematogenous spread in 15% to 30% of cases; and third, from a direct inoculation such as a head trauma or neurosurgery in 8% to 19% of cases. The origin of brain abscess formation remains unknown (cryptogenic brain abscess) in 20% to 30% of cases.^7^

The widespread and early use of antibiotics has resulted in decrease in incidence of brain abscess and clinical sequelae. Commonly identified risk factors include advanced age, cancer, chronic liver disease, diabetes mellitus, stroke, inflammatory bowel disease, HIV/AIDS, chronic obstructive pulmonary disease, and hemodialysis-dependent renal failure. Our patient lacked any of these risk factors. She was a young otherwise healthy woman without any of the identified risk factors. Her only past medical history was factor V Leiden mutation. After an extensive clinical workup, the presence of a definite source of the infection could not be identified. MRI findings of innumerable focal parenchymal lesions involving both cerebral hemispheres, deep gray matter, midbrain, and cerebellar hemispheres with distribution predominantly in the gray-white junctions supports a hematogenous spread.

The blood-brain barrier (BBB) is a selective barrier formed by the endothelial cells that line cerebral micro-vessels. It acts as a “physical barrier” because complex tight junctions between adjacent endothelial cells force most molecular traffic to take a transcellular route across the BBB rather than moving paracellularly through the junctions, as in most endothelia.^8^ The BBB and the blood-CSF barrier provide significant protection against microbes, creating a sterile environment for the CNS. An extensive literature review on our part did not yield any data or previous case reports of brain abscesses in context of factor V Leiden. However, antiphospholipid antibodies have an established association with macro- and microthrombosis as well as thromboembolic phenomena within the CNS. Autopsy studies from patients with APL antibodies have shown microinfarcts, microhemorrhages, and vasculopathy in in up to 80% cases. This shows that the even more diffuse CNS pathologies may have a vascular component and thus possible involvement of the BBB.^9^

Disruption in the BBB is a potential risk factor for hematogenous bacterial seeding of the brain parenchyma secondary to bacteremia. A history of factor V Leiden mutation in our patient, a prothrombotic state, may have resulted in increased permeability of the BBB. To the best of our knowledge, this is the first reported case of a serious CNS infection in an immunocompetent patient with factor V Leiden mutation, in the absence of established risk factors.

We therefore postulate a possible association of factor V Leiden mutation, a hypercoagulable state, causing microthrombi obstructing the small capillaries in BBB, resulting in microinfarctions and therefore increasing the permeability of microbes into the brain. More data are needed to confirm a true association.
